# Branched-Chain Amino Acids and Inflammation Management in Endurance Sports: Molecular Mechanisms and Practical Implications

**DOI:** 10.3390/nu17081335

**Published:** 2025-04-12

**Authors:** Miaomiao Xu, Danting Hu, Xiaoguang Liu, Zhaowei Li, Liming Lu

**Affiliations:** 1 School of Physical Education and Health, Guangzhou University of Chinese Medicine, Guangzhou 510006, China; miaomiaoxu@gzucm.edu.cn; 2 South China Research Center for Acupuncture and Moxibustion, Medical College of Acu-Moxi and Rehabilitation, Guangzhou University of Chinese Medicine, Guangzhou 510006, China; 20231110459@stu.gzucm.edu.cn; 3 College of Sports and Health, Guangzhou Sport University, Guangzhou 510500, China

**Keywords:** branched-chain amino acids, endurance athletes, muscle recovery, inflammation management, exercise-induced muscle damage

## Abstract

Endurance athletes frequently experience muscle damage and inflammation due to prolonged, high-intensity exercise, which can impair recovery and hinder performance. This review examines the role of branched-chain amino acid (BCAA) supplementation in muscle repair, inflammation modulation, and immune regulation. BCAAs—particularly leucine and isoleucine—activate key molecular pathways, including the mechanistic target of rapamycin (mTOR) and AMP-activated protein kinase (AMPK), to promote muscle protein synthesis and enhance energy metabolism. They also attenuate inflammatory responses by modulating the nuclear factor kappa-light-chain-enhancer of activated B cells (NF-κB), mitogen-activated protein kinase (MAPK), and Janus kinase/signal transducer and activator of transcription (JAK/STAT) pathways, reducing levels of tumor necrosis factor-alpha (TNF-α) and interleukin-6 (IL-6). In addition, BCAAs influence immune function via mechanistic target of rapamycin complex 1 (mTORC1) signaling, enhance autophagy, and mitigate exercise-induced apoptosis. These molecular effects result in reduced muscle soreness, lower muscle damage biomarker levels (e.g., creatine kinase, lactate dehydrogenase), and improved recovery. Practical considerations such as optimal dosage, timing, and co-supplementation with carbohydrates, proteins, or omega-3s are also addressed. While BCAAs show promise as a nutritional strategy for enhancing recovery and controlling inflammation in endurance athletes, further research is needed to refine personalized protocols and clarify long-term effects.

## 1. Introduction

Endurance athletes engage in prolonged and high-intensity training sessions, which can result in significant muscle damage, fatigue, and inflammatory responses [1,2]. Efficient recovery from such physiological stress is crucial not only for optimizing performance but also for reducing the risk of overtraining and injuries [3,4]. While a balanced diet remains the foundation of recovery nutrition, many athletes incorporate dietary supplements to enhance adaptation and performance [5,6]. Among these, branched-chain amino acids (BCAAs)—comprising leucine, isoleucine, and valine—are widely utilized for their potential benefits in muscle repair, soreness reduction, and immune modulation [7,8,9].

BCAAs are recognized for stimulating muscle protein synthesis [7,10], but it is important to distinguish between different types of protein synthesis relevant to recovery. Contractile protein synthesis involves the production of structural proteins like actin and myosin, which are essential for muscle function and repair after mechanical damage [10,11]. Conversely, cytoplasmic protein synthesis includes the production of enzymes and proteins that are essential for cellular metabolism, stress responses, and immune signaling [12,13], all of which are vital for restoring cellular homeostasis following endurance exercise [14]. BCAAs influence both types of protein synthesis, with leucine in particular playing a key role in activating the mechanistic target of rapamycin (mTOR) pathway. This pathway promotes the accretion of contractile proteins while also supporting cytoplasmic remodeling processes that help mitigate stress and inflammation [15,16].

Prolonged endurance activity triggers an inflammatory response involving cytokine release, oxidative stress, and immune activation, all of which can impair muscle regeneration and hinder subsequent performance [17]. BCAAs may offer a viable strategy for improving post-exercise recovery by modulating these inflammatory pathways. While numerous studies have investigated the role of BCAAs in muscle protein metabolism, research specifically linking BCAA supplementation to inflammation control in endurance athletes is limited. A review of available studies reveals only a few studies directly examining the effects of BCAAs on inflammation regulation in this population. This gap in the literature presents an opportunity to explore the potential mechanisms by which BCAAs may influence key inflammatory mediators and impact endurance performance.

At the molecular level, muscle damage from endurance exercise activates signaling cascades such as the mTOR and AMP-activated protein kinase (AMPK) pathways, both of which regulate protein synthesis and energy metabolism [18,19,20]. BCAAs not only serve as substrates for muscle protein synthesis but also modulate these pathways—particularly mTOR—to enhance muscle repair and potentially mitigate inflammatory responses [7,8,9,20]. Through mTOR activation, BCAAs help reduce inflammation by suppressing the production of pro-inflammatory cytokines and decreasing oxidative stress. Mechanistic target of rapamycin complex 1 (mTORC1) activation also inhibits the nuclear factor kappa B (NF-κB) pathway, which is responsible for initiating inflammation in response to muscle damage [7,8,9,20]. Additionally, BCAAs activate AMPK, which improves energy balance during recovery and further reduces inflammation by inhibiting inflammatory signaling pathways such as NF-κB and mitogen-activated protein kinase (MAPK). In addition, their effects on AMPK activity may enhance energy efficiency during recovery. Recent findings also suggest that BCAAs influence autophagy and apoptosis regulation in skeletal muscle cells [21,22]. Autophagy, a cellular process essential for clearing damaged components, is crucial after intense exercise to maintain tissue homeostasis [23]. BCAAs have been shown to modulate autophagic flux, potentially enhancing cellular repair mechanisms and promoting adaptation [24]. They also regulate apoptotic pathways by balancing pro- and anti-apoptotic protein expression, thereby preserving muscle integrity [25]. However, the relevance of these findings in endurance athletes remains largely unexplored, highlighting another area requiring further investigation.

This narrative review synthesizes current evidence on the role of BCAAs in inflammation management and recovery for endurance athletes. By evaluating both molecular mechanisms and practical applications, this review aims to clarify the extent to which BCAA supplementation can aid post-exercise recovery and inflammation control. In doing so, we seek to address the existing knowledge gap and provide evidence-based recommendations for optimizing BCAA use in endurance sport recovery strategies.

## 2. Methods

This narrative review was conducted to evaluate the effects of BCAAs on inflammation management and recovery in endurance athletes. A comprehensive literature search was performed using databases including PubMed, Scopus, and Web of Science, covering studies published between January 2018 and March 2025. The specific search strings used for each database included: PubMed: (“BCAAs” OR “branched-chain amino acids”) AND (“endurance exercise” OR “muscle recovery” OR “inflammation” OR “cytokine regulation”), Scopus: (“BCAAs” OR “branched-chain amino acids”) AND (“endurance exercise” OR “muscle recovery” OR “inflammation” OR “cytokine regulation”), and Web of Science: (“BCAAs” OR “branched-chain amino acids”) AND (“endurance exercise” OR “muscle recovery” OR “inflammation” OR “cytokine regulation”). Keywords included “BCAAs”, “endurance exercise”, “muscle recovery”, “inflammation”, and “cytokine regulation”.

Studies were included if they met the following criteria: (1) investigated the effects of BCAAs on muscle recovery, inflammation, or fatigue; (2) involved endurance-trained athletes or physically active individuals; and (3) were published in peer-reviewed journals.

Exclusion criteria: Studies were excluded if they met any of the following conditions: (1) non-English language publications; (2) studies focusing exclusively on strength training populations; (3) studies without clear BCAA supplementation protocols or outcomes related to inflammation or muscle recovery; and (4) studies that did not include relevant inflammatory markers or recovery outcomes.

**Figure 1:**
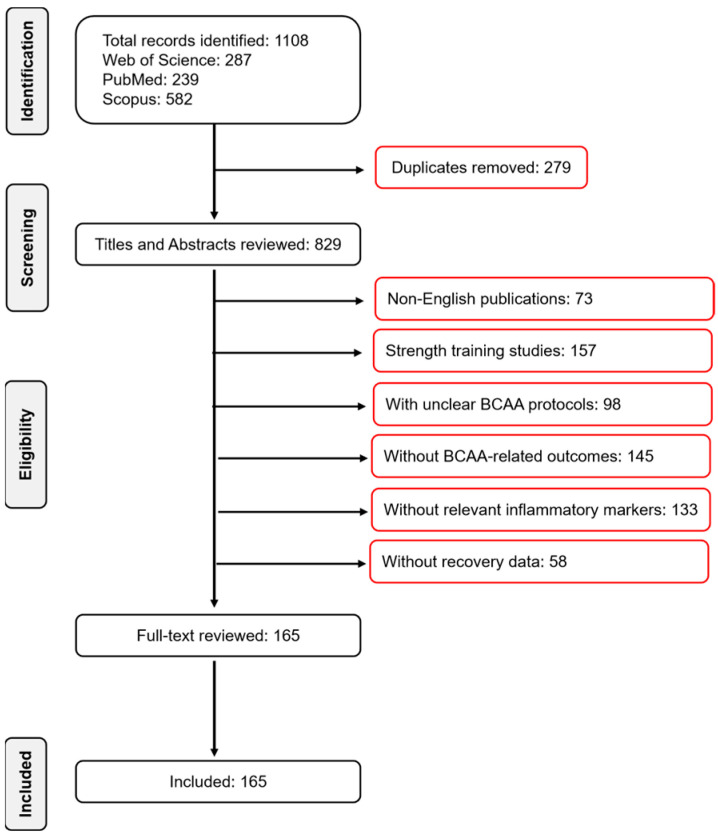
Literature selection flow diagram.

A total of 165 articles were identified and screened based on relevance and inclusion criteria. The screening process involved an initial review of titles and abstracts, followed by full-text screening to confirm eligibility. Out of the 165 articles, 120 full-text articles were assessed for eligibility, and 30 articles were ultimately included in the narrative synthesis. A PRISMA-like flow diagram outlining the study selection process is provided below to increase transparency (Figure 1).

## 3. Exercise-Induced Muscle Damage (EIMD) and Inflammation in Endurance Athletes

### 3.1. Muscle Damage and Inflammation Response

Endurance athletes frequently experience muscle damage and inflammation following prolonged or intense training sessions [26]. EIMD results from a combination of mechanical strain [27], oxidative stress [28,29], and metabolic disturbances [30], all of which compromise muscle fiber integrity and trigger an inflammatory response. Eccentric muscle contractions, in particular, cause microtears in muscle tissue that initiate the release of pro-inflammatory cytokines such as TNF-α and IL-6, both of which are essential in the early stages of tissue repair [31]. Concurrently, oxidative stress generates reactive oxygen species (ROS) that further damage cellular components [32], while metabolic byproducts like lactate exacerbate cellular disruption and delay recovery [33].

**Figure 2:**
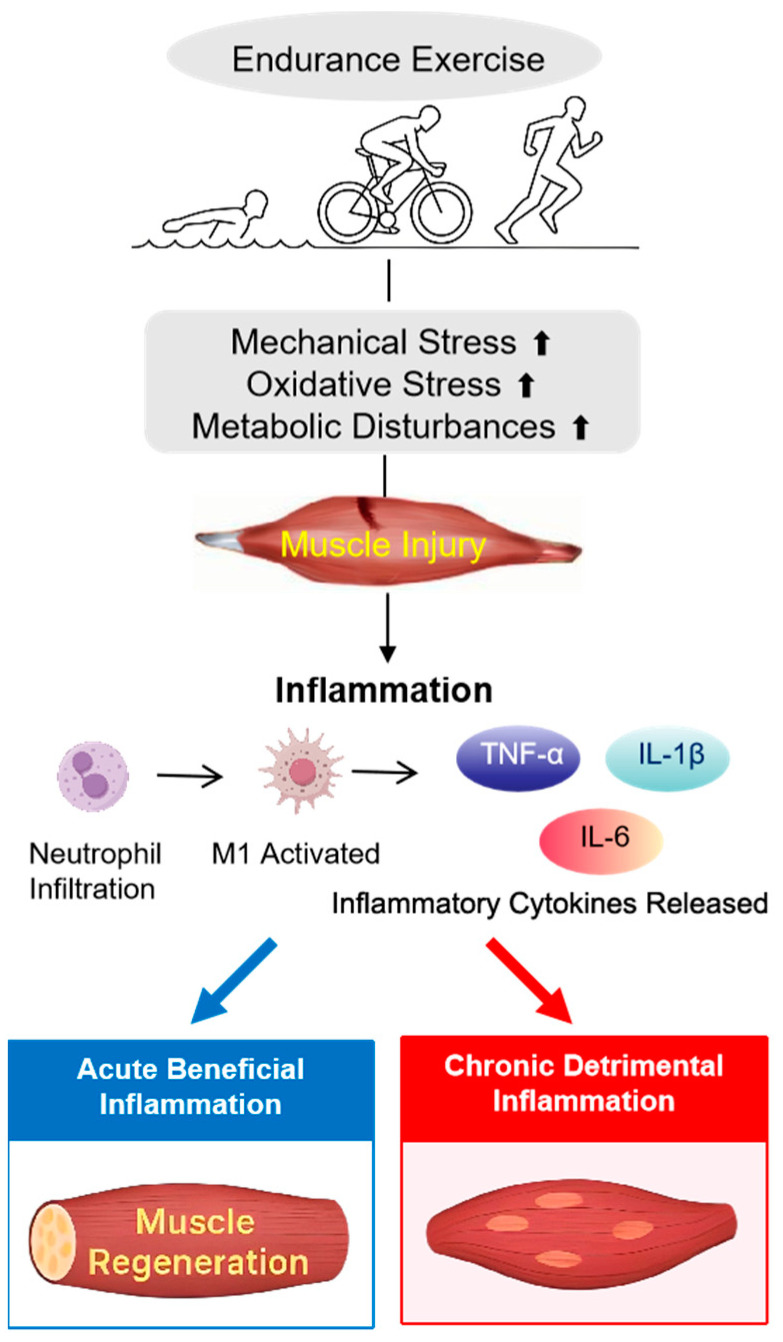
Mechanisms of muscle damage and inflammation. This schematic illustrates the processes by which endurance exercise, including activities like swimming, cycling, and running, induces muscle damage and inflammation. The physical stress from these activities results in mechanical and oxidative stress, which activates inflammation through the release of cytokines (IL-6, TNF-α, and interleukin-1 beta (IL-1β)). A moderate, acute inflammatory response (blue arrow) supports recovery and muscle regeneration, while excessive, chronic inflammation (red arrow) can impede recovery and negatively affect performance.

This cascade of biochemical and mechanical events activates a local and systemic inflammatory response. Although inflammation is an essential component of muscle repair and regeneration, excessive or prolonged inflammation may hinder tissue recovery and compromise future performance [34]. Thus, modulating the inflammatory response following endurance exercise is vital for optimizing recovery and minimizing performance deficits (Figure 2).

### 3.2. Physiological Mechanisms of EIMD

EIMD is primarily driven by the mechanical strain of eccentric contractions. This strain disrupts the sarcomere structure and leads to muscle fiber damage [35,36]. This structural damage triggers a coordinated inflammatory response involving multiple signaling molecules and immune cell recruitment (Table 1). Among the central mediators, IL-6 plays a dual role by facilitating both inflammatory signaling and subsequent muscle repair and adaptation processes [37,38,39]. In contrast, cytokines such as TNF-α and IL-1β promote inflammation and, when elevated beyond a threshold, contribute to secondary tissue damage [39,40,41,42].

In addition, chemokines such as monocyte chemoattractant protein-1 (MCP-1) and interleukin-8 (IL-8) are upregulated, recruiting neutrophils and macrophages to the injury site to clear debris and initiate repair [42]. CRP, a marker of systemic inflammation, is also commonly elevated after intense exercise and reflects the extent of immune activation [32,43,44,45].

On a molecular level, these cytokines activate the NF-κB signaling pathway, a key transcriptional regulator of inflammatory gene expression [46,47]. Additionally, exercise-induced oxidative stress promotes the formation of ROS, which not only exacerbate tissue damage but also interfere with cellular recovery processes by modulating apoptotic and autophagic pathways [48,49].

**Table 1:** Key inflammatory markers post-exercise.

Inflammatory Marker	Description	Role Post-Exercise	Normal/Baseline Values	Typical Changes Post-Exercise	References
IL-6	Cytokine involved in immune response and inflammation	Released during exercise; promotes muscle repair and adaptation	~1–5 pg/mL	Increases post-exercise, peaks within 1–2 h, returns to baseline after a few hours	Fernández-Lázaro et al. [40]
TNF-α	Pro-inflammatory cytokine	Induces inflammation; excessive levels can cause muscle damage	~1–3 pg/mL	Peaks 1–3 h post-exercise, elevated for several hours	Mallett et al. [29]
CRP	Protein produced by the liver in response to inflammation	Marker of systemic inflammation; elevated after intense exercise	~1–3 mg/L	Significant increase post-exercise, peaks after 24 h	Zhao et al. [50]
IL-1β	Pro-inflammatory cytokine	Plays a role in tissue damage response and muscle inflammation	~2–5 pg/mL	Increases immediately post-exercise, returns to baseline within 24–48 h	Notbohm et al. [37]
MCP-1	Chemokine that attracts immune cells to inflammation sites	Mediates macrophage recruitment to injured muscle tissue	~100–300 pg/mL	Increases 2–6 h post-exercise, declines within 24 h	Lagzdina et al. [51]
IL-8	Chemokine involved in attracting neutrophils to inflammation sites	Facilitates neutrophil recruitment; supports repair and inflammation	~5–10 pg/mL	Peaks 2–4 h post-exercise, returns to baseline within 24 h	Małkowska et al. [42]

TNF-α, Tumor Necrosis Factor-alpha; IL-6, Interleukin-6; CRP, C-Reactive Protein; IL-1β, Interleukin-1 beta; MCP-1, Monocyte Chemoattractant Protein-1; IL-8, Interleukin-8.

### 3.3. Inflammatory Response and Recovery

While acute inflammation is essential for muscle regeneration, uncontrolled or chronic inflammation can impair recovery and muscle function [51,52,53]. The post-exercise inflammatory process occurs in three sequential phases:

(1) Acute Inflammatory Phase—Neutrophils activate M1 macrophages, which release pro-inflammatory cytokines (TNF-α, IL-1β, IL-6) to initiate the repair cascade [54];
(2) Resolution Phase—A shift occurs toward M2 macrophage activity, characterized by anti-inflammatory cytokines such as interleukin-10 (IL-10) and transforming growth factor beta (TGF-β), which promote healing and regeneration [55];
(3) Regeneration Phase—Satellite cells are activated to repair and remodel muscle fibers [2,56].

**Figure 3:**
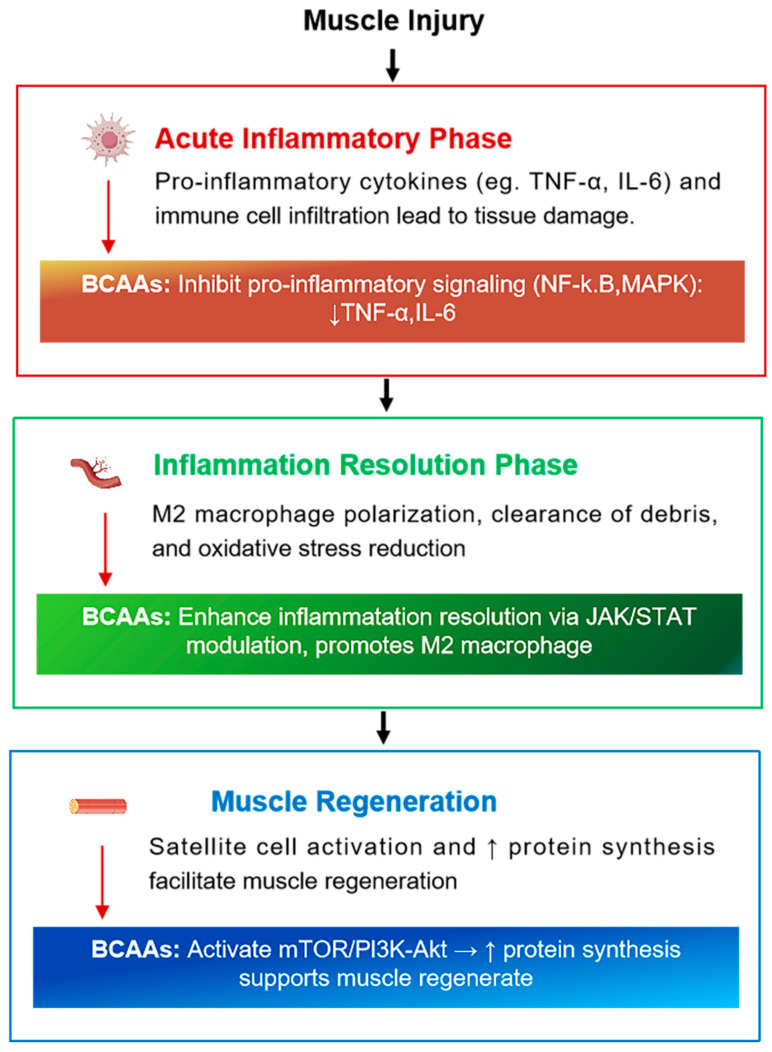
Inflammatory pathways in muscle repair and BCAAs’ functions. This figure illustrates the sequential inflammatory phases involved in muscle repair following exercise-induced damage. The Acute Inflammatory Phase involves neutrophil infiltration and activation of M1 macrophages, which release pro-inflammatory cytokines (TNF-α, IL-6). During the Inflammation Resolution Phase, a phenotypic shift to M2 macrophages occurs, promoting anti-inflammatory cytokine release (e.g., IL-10, TGF-β) and tissue remodeling. The final Muscle Regeneration Phase features satellite cell activation and myofiber repair. BCAAs modulate these phases by inhibiting NF-κB/MAPK signaling, enhancing Janus kinase/signal transducer and activator of transcription (JAK/STAT)-mediated M2 macrophage polarization, and activating the mTOR/phosphatidylinositol 3-kinase (PI3K)/protein kinase B (Akt) pathway to support protein synthesis and regeneration.

However, when the inflammatory response remains elevated beyond the acute phase, it can impair muscle regeneration, increase the risk of muscle soreness and injury, and negatively impact subsequent performance [57]. Therefore, regulating the magnitude and duration of inflammation is crucial. Nutritional interventions such as BCAA supplementation may help balance the inflammatory process, promoting effective muscle recovery without impairing natural adaptive responses [6,58,59] (Figure 3).

## 4. Role of BCAAs in Muscle Recovery

### 4.1. Mechanisms of Action

BCAAs play a crucial role in muscle recovery by promoting protein synthesis and reducing muscle breakdown [8]. Among the three BCAAs—leucine, isoleucine, and valine—leucine is particularly potent due to its ability to activate the mTORC1 signaling pathway [15,60]. The activation of mTORC1 enhances protein synthesis by promoting translation initiation and upregulating ribosomal activity, thereby accelerating the repair of damaged muscle fibers [61,62]. Leucine interacts with Rag GTPases, facilitating the translocation of mTORC1 to the lysosomal membrane and activating downstream effectors such as S6K1 and 4E-BP1 [63,64].

Furthermore, BCAAs inhibit muscle protein degradation by suppressing the ubiquitin–proteasome system, thereby helping to preserve muscle mass [65,66]. This is achieved by downregulating forkhead box O (FoxO) transcription factors and reducing the expression of ubiquitin ligases, both of which contribute to decreased proteolysis [66]. Among all three BCAAs, leucine’s dual anabolic and anti-catabolic roles make it a critical modulator of post-exercise muscle recovery and adaptation [16,67,68].

In addition to protein turnover, BCAAs influence insulin signaling and enhance insulin sensitivity, thereby facilitating glucose uptake and glycogen replenishment in muscle tissue [69,70], This metabolic role is particularly valuable following endurance exercise, when glycogen stores are depleted [6,71,72].

BCAAs also serve as a supplemental energy source during prolonged or high-intensity exercise [73], thereby sparing glycogen reserves, reducing fatigue [74], and helping to sustain performance [75], which is of particular relevance to endurance athletes [76]. Furthermore, BCAAs help preserve muscle mass during caloric restriction or extreme training loads by limiting protein breakdown [8,77].

Another critical mechanism is BCAAs’ ability to mitigate central fatigue. By competing with tryptophan for transport across the blood–brain barrier, BCAAs reduce serotonin production [78,79], a neurotransmitter associated with perceived fatigue [80]. Lower serotonin levels have been linked to improved focus and extended performance capacity, making this effect especially valuable in endurance sports [81,82].

Together, these mechanisms highlight BCAAs as multifunctional agents that enhance protein synthesis, reduce catabolism, support energy metabolism, and limit fatigue—key components of effective recovery in endurance athletes (Table 2).

**Table 2:** Comparison of BCAAs’ roles in protein synthesis.

BCAA	Effect on Protein Synthesis	Mechanism of Action	Relative Potency/Optimal Ratio	References
Leucine	Strongly stimulates protein synthesis, activates mTOR pathway	Activates mTORC1 signaling pathway, increasing protein synthesis rate	Highest potency for protein synthesis; typically found in a 2:1:1 ratio with isoleucine and valine	Ma et al. [67]
Isoleucine	Promotes muscle protein synthesis, weaker mTOR activation effect	Increases energy availability, promotes protein synthesis, involved in muscle metabolism regulation	Moderate potency; often combined with leucine and valine in a 2:1:1 ratio to enhance effects	Mao et al. [83]
Valine	Weaker effect on protein synthesis	Regulates amino acid balance, influences synthesis of other amino acids	Least potent for protein synthesis; part of the 2:1:1 ratio for optimal effect	Beigi et al. [84]

### 4.2. BCAAs and Muscle Damage

BCAA supplementation has been extensively studied for its role in reducing muscle damage, with consistent findings showing lower post-exercise levels of biomarkers such as creatine kinase (CK) and lactate dehydrogenase (LDH) [7,85]. These biomarkers reflect membrane disruption and metabolic stress, and reductions indicate improved muscle integrity and reduced tissue damage [86].

By promoting protein synthesis and reducing proteolysis, BCAAs exert a dual protective effect—supporting both structural maintenance and tissue repair [8]. They also reduce delayed-onset muscle soreness and enhance regeneration, benefits that are particularly relevant for endurance athletes who are subject to repeated physical stress [87,88,89,90]. Timely post-exercise BCAA intake has been linked to faster recovery and sustained training output [58,71,91].

Emerging evidence also suggests antioxidant properties for BCAAs, which may reduce oxidative-stress-related muscle damage. Specifically, BCAAs help regulate the nicotinamide adenine dinucleotide (NAD^+^/NADH) ratio, thereby supporting mitochondrial function and limiting ROS accumulation [92,93]. By promoting the cellular redox balance, BCAAs contribute to protecting muscle cells from oxidative injury and maintaining metabolic homeostasis [22,92].

Recent research has highlighted the role of the sirtuin 1 (SIRT1) axis—a key cellular regulator involved in oxidative stress response, mitochondrial biogenesis, and metabolic homeostasis—in muscle recovery [94,95]. SIRT1 is a nicotinamide adenine dinucleotide (oxidized form)-dependent deacetylase that modulates the activity of transcription factors such as peroxisome proliferator-activated receptor gamma coactivator 1-alpha (PGC-1α) and FOXO, promoting antioxidant defense and mitochondrial function [94]. Given that BCAA metabolism affects the NAD^+^/NADH ratio, it may also influence SIRT1 activity [22,96]. Through this mechanism, BCAAs may indirectly activate SIRT1 signaling, enhancing cellular resilience to oxidative stress and supporting more efficient recovery in endurance athletes [97]. Although direct evidence remains limited, the potential interaction between BCAAs and the SIRT1 axis represents a promising area for further investigation [22].

Taken together, these findings position BCAAs as a valuable tool in minimizing muscle damage, accelerating repair, and potentially engaging protective pathways such as the SIRT1 axis to support recovery in endurance training scenarios.

### 4.3. BCAAs and Fatigue Reduction

Fatigue is a common challenge for endurance athletes, affecting both performance and recovery [98,99]. One major contributor to fatigue is the accumulation of serotonin in the brain, which promotes sensations of tiredness and reduced motivation during prolonged exercise [80,100]. BCAAs, particularly leucine, help counteract this effect by competing with tryptophan for transport across the blood–brain barrier, thereby limiting serotonin synthesis and reducing the onset of central fatigue [101,102].

By lowering serotonin availability, BCAAs enable athletes to maintain higher cognitive alertness, motivation, and neuromuscular efficiency throughout extended activity [103]. This central effect complements the peripheral roles of BCAAs in muscle metabolism, making them uniquely positioned to combat fatigue on multiple fronts.

Peripheral fatigue mechanisms: In addition to modulating neurotransmitter activity, BCAAs delay fatigue by preserving glycogen stores and serving as an alternative energy substrate during endurance exercise [104]. This energy-sparing effect allows athletes to sustain higher intensities for longer durations, improving both training quality and competition performance [105,106,107]. The ability to buffer against early fatigue not only enhances athletic output but also contributes to better training adaptations over time [108].

Central fatigue mechanisms: BCAAs have also been associated with reduced perceptions of effort and improved mood state during high-stress physical activity, likely due to their impact on central nervous system fatigue mechanisms [79,109]. By modulating brain neurotransmitter levels, particularly the balance between serotonin and dopamine, BCAAs help maintain mental sharpness and psychological resilience during endurance events [110,111,112].

Regular BCAA supplementation during periods of high training load or competition may also attenuate exercise-induced muscle soreness, reduce systemic inflammation, and further support recovery [59,113]. In doing so, BCAAs offer a comprehensive approach to fatigue management—addressing both peripheral energy systems and central fatigue regulation (Table 3).

**Table 3:** Fatigue reduction mechanism of BCAAs.

BCAA	Effect on Fatigue	Mechanism of Action	References
Leucine	Reduces central fatigue and improves endurance performance	Inhibits serotonin production in the brain by competing with tryptophan for transport into the brain	Wen et al. [114]
Isoleucine	Enhances energy production and reduces fatigue during prolonged exercise	Increases glucose uptake into muscles, enhancing energy availability and reducing perceived fatigue	Baraniuk et al. [115]
Valine	Reduces perceived exertion and delays fatigue onset	Competes with tryptophan to lower serotonin levels, reducing central fatigue during prolonged exercise	Duttagupta et al. [116]

## 5. BCAAs and Inflammation Management in Endurance Athletes

### 5.1. BCAAs Modulating Inflammatory Cytokines

BBCAAs, particularly leucine and isoleucine, have been shown to modulate inflammation by regulating cytokine expression [83,117]. Figure 3 provides an overview of how BCAAs modulate key inflammatory signaling pathways during muscle repair following exercise-induced damage. Intense endurance exercise often leads to increased levels of pro-inflammatory cytokines such as TNF-α and IL-6, which are associated with muscle damage and delayed recovery [35]. Elevated concentrations of these cytokines can prolong the inflammatory response, exacerbate tissue breakdown, and impair adaptation [56,118].

Quantitative data suggest that BCAA supplementation can significantly reduce the release of these inflammatory cytokines. For example, studies have shown that BCAA intake can lower TNF-α levels by approximately 20–30% and IL-6 levels by 15–25% post-exercise [58,119]. This reduction in cytokine release helps dampen the immune response and supports faster muscle regeneration [58]. By lowering TNF-α and IL-6 levels, BCAAs may help athletes recover more efficiently and maintain training quality during periods of repeated exertion [117]. Additionally, minimizing inflammation may enhance the athlete’s ability to adapt to training without excessive tissue stress, reducing the likelihood of overtraining or injury [120].

At the molecular level, BCAAs—especially leucine and isoleucine—modulate the JAK/STAT signaling pathway, which plays a key role in cytokine signal transduction [83,117]. Endurance exercise activates inflammatory cascades through the NF-κB and MAPK pathways, both of which drive cytokine expression [35]. BCAAs appear to inhibit excessive activation of these inflammatory pathways, limiting downstream cytokine release and immune cell infiltration [117].

Furthermore, BCAAs influence the PI3K/Akt pathway, which governs cell survival and anti-apoptotic signaling [119]. By enhancing Akt activation and reducing pro-apoptotic stress, BCAAs may help protect muscle cells from inflammation-induced damage and preserve functional muscle tissue [121].

### 5.2. Immune System Regulation

In addition to their effects on inflammation, BCAAs contribute to immune system maintenance, especially following strenuous exercise [122,123]. Endurance activity can transiently suppress immune function, leading to reductions in lymphocyte activity and increased susceptibility to infection [124,125,126,127]. BCAA intake has been linked to improved T-cell proliferation and enhanced immune surveillance, helping athletes maintain resilience during high training loads [128,129,130,131,132] (Table 4).

BCAAs also play a role in immune cell metabolism, acting as fuel for rapidly proliferating cells and supporting their biosynthetic needs [128]. By activating the mTORC1 pathway in immune cells, BCAAs promote cell growth, differentiation, and cytokine production, all of which are essential for effective immune defense [128]. These effects are particularly relevant in the post-exercise period, when immune recovery is essential to support tissue regeneration and defend against pathogens.

BCAAs also help reduce inflammation [133,134], maintaining a balanced immune response [120,135]. By boosting immune cell activity and reducing inflammation, BCAAs enhance resilience to infections and support faster recovery [136]. This allows endurance athletes to train consistently while minimizing illness risk [137], benefiting both muscle recovery and overall immune health.

BCAAs not only play an important role in muscle function but are also critical for maintaining immune system health [122,123]. After intense endurance exercise, the immune system can become temporarily suppressed, leading to lower lymphocyte counts and an elevated risk of infection [124,125,126]. By enhancing the metabolic activity and proliferation of T-cells, BCAAs strengthen immune function [128,129,130,131,132]. Specifically, BCAAs activate the mTORC1 signaling pathway, promoting protein synthesis and cell division in T-cells, thereby improving the efficiency of immune responses [128]. Furthermore, BCAAs regulate inflammatory pathways, helping maintain immune system balance and preventing tissue damage caused by excessive inflammation [133,134].

**Table 4:** Effects of BCAAs on immune function.

BCAA	Effect	Impact on Immune Function	References
Leucine, Isoleucine, and Valine	Immune Cell Proliferation	Stimulates immune cell growth	Yahsi et al. [138]
Leucine	Cytokine Production	Increases production of IL-6 and TNF-α	Brown et al. [117]
All BCAAs	T-Cell Activation	Enhances T-cell activation	Yao et al. [130]
Isoleucine	Macrophage Function	Modulates macrophage activity	Mao et al. [83]
Valine	Inflammation	Reduces chronic inflammation	Gart et al. [139]
Leucine and Isoleucine	Overall Immune Response	Improves immune response during stress	Wen et al. [114]

### 5.3. Research Evidence on Inflammation Management

Several studies support the role of BCAA supplementation in reducing inflammation and improving recovery outcomes in endurance athletes [120,140,141]. For example, BCAAs have been shown to lower post-exercise serum IL-6 levels, indicating reduced systemic inflammation and stress [142]. These anti-inflammatory effects may contribute to faster recovery, reduced soreness, and improved performance during subsequent training sessions [7,91,140].

Additionally, BCAAs appear to affect innate immune regulation by modulating inflammasome activity—specifically the NLRP3 complex—which triggers IL-1β and interleukin-18 release [143]. By inhibiting inflammasome assembly and activation, BCAAs help prevent excessive cytokine release, reducing chronic inflammation and promoting a balanced immune response [142].

In terms of endurance training recovery, BCAAs have a critical role in immune function, particularly in enhancing T-cell activity. BCAA supplementation has been shown to promote T-cell proliferation and differentiation, which are essential for maintaining immune surveillance and defending against pathogens during the recovery phase [130]. This enhanced immune function supports faster recovery by improving tissue regeneration and reducing the risk of infections that can disrupt the recovery process [6]. By bolstering the immune system, BCAAs also help prevent exercise-induced immune suppression, allowing athletes to maintain a higher level of resilience during periods of intense or frequent endurance training [76].

In combination, these findings demonstrate that BCAAs provide a valuable means of controlling inflammation and supporting immune function during high-volume endurance training. Their capacity to regulate cytokine production, protect against immune suppression, and promote tissue recovery makes them a promising nutritional intervention for athletes facing intense or frequent training stress [9].

## 6. Practical Considerations for BCAA Supplementation in Endurance Athletes

### 6.1. Dosage and Timing

To maximize the physiological benefits of BCAAs, a daily intake of 5 to 20 g has been recommended based on current research [76,81,90]. However, the optimal dosage for endurance athletes varies depending on factors such as body weight, training volume, and dietary protein intake. Higher doses may be beneficial during periods of intense training or competition, particularly when muscle recovery and inflammation management are critical.

The timing of supplementation is another key factor. Studies indicate that consuming BCAAs immediately post-exercise or even during prolonged endurance activity can enhance muscle protein synthesis and accelerate recovery [7,144].

Additionally, co-ingestion with carbohydrates has been shown to enhance BCAA uptake and utilization [76,91,145]. This combination promotes insulin secretion, facilitating amino acid transport into muscle cells and thereby enhancing glycogen replenishment and muscle repair [145]. Such synergistic effects suggest that BCAAs should be considered within a broader nutritional strategy rather than as an isolated intervention (Table 5).

**Table 5:** BCAA supplementation recommendations by athlete profile.

Athlete Profile	BCAA Dosage (Grams per Day)	Timing Options	Comments	Study Type	References
Ultra-marathon runners	5–10 g	Pre- and post-race	Reduces muscle soreness, speeds recovery by 20%	Observational study	Zhang et al. [146]
Triathletes	15 g	Post-training	Decreases muscle damage markers by 25%	Randomized controlled trial	Durkalec-Michalski et al. [147]
Swimmers	18–20 g	Pre- and post-training	Improves recovery, reduces fatigue by 18%	Cross-sectional study	Cai et al. [148]
Cyclists	84 mg/kg	During training	Improves endurance, reduces fatigue during sessions	Randomized controlled trial	Fa et al. [149]

### 6.2. Synergistic Effects with Other Supplements

BCAAs exert greater benefits when combined with other key nutritional components, including proteins, carbohydrates, and antioxidants [102,150]. Protein supplementation provides essential amino acids that complement the effects of BCAAs in muscle repair, while carbohydrates replenish glycogen stores depleted during exercise [145,151]. Additionally, antioxidants mitigate oxidative stress, which is often heightened during endurance activity, further supporting recovery [152].

**Figure 4:**
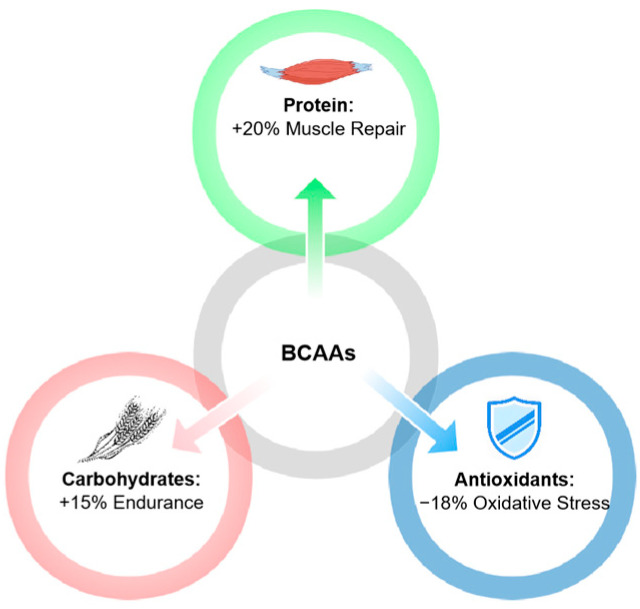
Synergistic effects of BCAAs with other supplements. This figure shows how BCAAs work synergistically with protein, carbohydrates, and antioxidants to enhance recovery and performance. When combined with protein, BCAAs improve muscle repair by 20%; paired with carbohydrates, they increase endurance by 15% through glycogen replenishment; antioxidants reduce oxidative stress by 18%, minimizing muscle damage and speeding up recovery. The arrows indicate the interactions between BCAAs and each supplement.

Research has shown that combining BCAAs with protein enhances muscle repair by up to 20% compared to BCAAs alone [16]. Similarly, co-ingestion of carbohydrates has been linked to a 15% improvement in endurance performance through more efficient glycogen resynthesis [3]. Antioxidants, such as vitamin C and E, have been reported to reduce markers of oxidative stress by approximately 18%, contributing to faster recovery and reduced muscle soreness [153] (Figure 4).

Furthermore, BCAAs in combination with omega-3 fatty acids have demonstrated enhanced anti-inflammatory effects. Omega-3s influence cell membrane fluidity and receptor function, potentially amplifying the anabolic and recovery-promoting effects of BCAAs [150,154]. Optimal Ratios: For maximal synergistic effects, studies suggest the following optimal ratios:

BCAAs to Protein: A 2:1 ratio of BCAAs to protein is often recommended for enhanced muscle repair and recovery [16].

BCAAs to Carbohydrates: A 1:3 ratio of BCAAs to carbohydrates has been shown to improve glycogen resynthesis and endurance performance [3].

BCAAs to Omega-3 Fatty Acids: A ratio of 3:1 for BCAAs to omega-3 fatty acids may provide the most significant anti-inflammatory and recovery benefits [150].

This suggests that a multi-component supplementation approach, with carefully balanced ratios, may yield superior recovery and performance benefits.

### 6.3. Application

From a practical perspective, BCAA supplementation offers benefits for endurance athletes under several conditions, including high-intensity training periods, prolonged events, and recovery from muscle fatigue or injury [149]. Athlete-reported benefits and controlled trials suggest that BCAA intake reduces muscle soreness, lowers post-exercise inflammation, and improves recovery markers such as CRP and IL-6 levels [58,155].

Several case studies highlight the efficacy of BCAA supplementation in endurance athletes (Table 5). For example, athletes undergoing intense marathon training have reported reduced perceptions of muscle fatigue and improved recovery when incorporating BCAAs into their post-exercise nutrition [81]. Similarly, triathletes supplementing with BCAAs have demonstrated lower post-exercise CK and LDH levels, indicative of reduced muscle damage [81]. Such findings reinforce the practical benefits of BCAAs in endurance sports settings.

Different endurance disciplines may require tailored BCAA supplementation approaches. For instance, long-distance runners and cyclists, who experience prolonged muscle catabolism, may benefit from intra-exercise BCAA intake to sustain performance and delay fatigue [151]. In contrast, rowers or swimmers, who engage in repeated high-intensity efforts, might prioritize BCAA consumption post-exercise to accelerate muscle repair and limit inflammatory responses [156]. Developing sport-specific supplementation protocols could optimize the benefits of BCAAs for endurance athletes.

Despite promising evidence, individual responses to BCAA supplementation may vary, influenced by baseline dietary intake, training status, and metabolic efficiency. Therefore, personalized strategies based on training demands and recovery needs should guide supplementation protocols. Additionally, combining BCAAs with other recovery-supporting nutrients may enhance their efficacy. For example, co-supplementation with omega-3 fatty acids, known for their anti-inflammatory properties, may further reduce exercise-induced muscle damage [150,157]. Likewise, integrating BCAAs with high-quality protein sources such as whey or casein can provide a more comprehensive amino acid profile, maximizing muscle protein synthesis and recovery [158]. These synergistic strategies warrant further exploration to establish evidence-based guidelines for endurance athletes.

## 7. Limitations and Future Directions

### 7.1. Limitations of Current Research

Despite promising evidence supporting the role of BCAA supplementation in endurance athlete recovery, several limitations exist within the current body of research. Variability in study design—including differences in BCAA dosage, supplementation duration, and participant demographics—make it difficult to establish universal recommendations. Many studies have focused on acute responses to BCAA supplementation, while the long-term effects on chronic inflammation, muscle adaptation, and overall performance remain insufficiently explored.

Furthermore, most research has relied on indirect biomarkers such as CK, LDH, and inflammatory cytokines to assess recovery outcomes, rather than utilizing direct measures of muscle repair, protein turnover, or performance recovery. Future studies employing muscle biopsies, molecular assays, and advanced imaging techniques could provide more comprehensive insights into the underlying mechanisms of BCAA action.

Another key limitation is the potential confounding effects of dietary intake and co-supplementation. Few studies have controlled for total protein and amino acid intake, making it difficult to determine whether the observed benefits of BCAA supplementation arise from their direct effects or simply from increased total protein availability. Future trials should standardize dietary intake and evaluate BCAA supplementation as part of a holistic nutritional approach.

Additionally, genetic and metabolic variability among athletes may influence individual responses to BCAA supplementation, yet this aspect has received limited attention. Emerging research on nutrigenomics and personalized nutrition suggests that genetic polymorphisms affecting BCAA metabolism could impact their efficacy in muscle recovery and inflammation management. Understanding these inter-individual differences may lead to more targeted and effective supplementation strategies.

### 7.2. Future Research Directions

To enhance our understanding of BCAA supplementation in endurance athletes, future research should focus on several key areas:

(1) Standardization of Dosage and Protocols—There is a need for large-scale, well-controlled trials to determine optimal BCAA dosages, ratios, and timing specific to endurance athletes. Personalized supplementation strategies should be explored based on training intensity, metabolic demands, and genetic predisposition. Methodological recommendations include the use of double-blind, placebo-controlled designs and the establishment of standardized protocols to account for variations in exercise modalities and recovery periods.
(2) Long-Term Effects on Recovery and Performance—While the short-term benefits of BCAA supplementation are well-documented, more research is needed to assess its long-term impact on muscle adaptation, chronic inflammation, and endurance capacity over extended training cycles. Future studies should implement longitudinal designs with repeated measures to capture changes over time and ensure more reliable conclusions about the long-term effects.
(3) Integration with Other Nutritional Strategies—Investigating the synergistic effects of BCAAs when combined with other nutrients (e.g., carbohydrates, protein, omega-3s, and antioxidants) could provide valuable insights into optimal recovery protocols. Methodologically, studies should employ a multi-variable approach, controlling for nutrient interactions, and utilize metabolomic and proteomic analyses to explore synergistic effects at the molecular level.
(4) Mechanistic Insights into Muscle and Immune Function—Advanced molecular and omics-based approaches (e.g., proteomics, metabolomics) should be employed to uncover the precise biochemical pathways through which BCAAs influence muscle repair, immune regulation, and mitochondrial function. It would be beneficial to combine these omics-based approaches with in vivo animal models and human trials to validate findings and provide a more comprehensive understanding of underlying mechanisms.
(5) Personalized Nutrition and Genetic Factors—Future research should examine how genetic variations in amino acid metabolism affect BCAA utilization and efficacy in different athlete populations. Such findings could pave the way for more individualized and evidence-based supplementation strategies. Studies should incorporate genetic screening alongside phenotypic assessments to create a more robust understanding of individual variability and its impact on BCAA supplementation outcomes.

By addressing these research gaps, future studies can establish more definitive guidelines for BCAA use in endurance sports and provide a clearer understanding of their role in recovery, inflammation management, and performance optimization.

## 8. Conclusions

BCAAs play a crucial role in endurance athlete recovery by enhancing muscle protein synthesis, reducing protein breakdown, and modulating inflammation. Their influence on key pathways such as mTORC1 and AMPK supports tissue repair, while their ability to regulate inflammatory cytokines, including TNF-α and IL-6, promotes a balanced immune response. Additionally, BCAAs help delay central fatigue by competing with tryptophan, limiting serotonin production, and sustaining cognitive and neuromuscular function.

Despite these benefits, further research is needed to refine dosage recommendations, assess long-term effects, and explore personalized supplementation strategies. Integrating BCAAs into a well-rounded nutritional approach may enhance recovery, performance, and resilience in endurance athletes.

## Data Availability

All relevant materials are presented in this manuscript.
